# 4,10-Bis(pyridin-2-ylmeth­yl)-1,7-dithia-4,10-diazo­niacyclo­dodecane bis(perchlorate)

**DOI:** 10.1107/S1600536811032715

**Published:** 2011-08-27

**Authors:** ZhenHong Wei, XiuLi You

**Affiliations:** a Department of Chemistry, Nanchang University, Nanchang 330031, People’s Republic of China

## Abstract

The asymmetric unit of the title compound C_20_H_30_N_4_S_2_^+^. 2ClO_4_^−^ comprises one macrocyclic cation and two perchlorate anions. In the cation, one of the protonated H atoms bound to the amide N atom is involved in an intra­molecular N—H⋯N hydrogen bond. The O atoms in the two perchlorate anions are disordered over two sets of sites with occupancy ratios of 0.65 (3):0.35 (3) and 0.640 (15):0.360 (15).

## Related literature

For tunable physicochemical and functional properties of macrocyclic ligands, see: Fabbrizzi *et al.* (1999 ▶). For applications of transition metal complexes with macrocyclic ligands, see: De Silva *et al.* (2003 ▶); Habata *et al.* (2006 ▶); Bilgin *et al.* (2009 ▶); Bernier *et al.* (2011 ▶). For similar structures, see: Peng *et al.* (2009 ▶); Wasitlewski & Mattes (1990 ▶); Funkemeier & Mattes (1993 ▶); Chak & McAuley (2006 ▶).
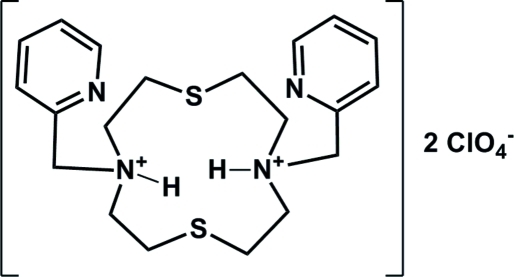


## Experimental

### 

#### Crystal data

C_20_H_30_N_4_S_2_^2+^·2ClO_4_^−^
*M* *_r_* = 589.52
Triclinic, 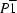

*a* = 10.4491 (6) Å
*b* = 11.6146 (7) Å
*c* = 11.7795 (7) Å
α = 96.167 (1)°
β = 90.340 (1)°
γ = 113.830 (1)°
*V* = 1298.23 (13) Å^3^
*Z* = 2
Mo *K*α radiation
μ = 0.46 mm^−1^
*T* = 296 K
0.35 × 0.30 × 0.25 mm

#### Data collection

Bruker APEXII CCD diffractometer
Absorption correction: multi-scan (*SADABS*; Sheldrick, 2003 ▶) *T*_min_ = 0.850, *T*_max_ = 0.891
12276 measured reflections
6516 independent reflections
4865 reflections with *I* > 2σ(*I*)
*R*_int_ = 0.018

#### Refinement

*R*[*F* ^2^ > 2σ(*F* ^2^)] = 0.041
*wR*(*F* ^2^) = 0.114
*S* = 1.04
6516 reflections
407 parameters
84 restraints
H atoms treated by a mixture of independent and constrained refinement
Δρ_max_ = 0.37 e Å^−3^
Δρ_min_ = −0.37 e Å^−3^

### 

Data collection: *APEX2* (Bruker, 2007 ▶); cell refinement: *SAINT* (Bruker, 2007 ▶); data reduction: *SAINT*; program(s) used to solve structure: *SHELXS97* (Sheldrick, 2008 ▶); program(s) used to refine structure: *SHELXL97* (Sheldrick, 2008 ▶); molecular graphics: *ORTEP-3 for Windows* (Farrugia, 1997 ▶); software used to prepare material for publication: *SHELXL97*.

## Supplementary Material

Crystal structure: contains datablock(s) I, global. DOI: 10.1107/S1600536811032715/jj2097sup1.cif

Structure factors: contains datablock(s) I. DOI: 10.1107/S1600536811032715/jj2097Isup2.hkl

Supplementary material file. DOI: 10.1107/S1600536811032715/jj2097Isup3.cml

Additional supplementary materials:  crystallographic information; 3D view; checkCIF report

## Figures and Tables

**Table 1:** Hydrogen-bond geometry (Å, °)

*D*—H⋯*A*	*D*—H	H⋯*A*	*D*⋯*A*	*D*—H⋯*A*
N2—H1*B*⋯N4	0.89 (2)	2.06 (2)	2.661 (3)	123.6 (19)

**Fig. 1.**
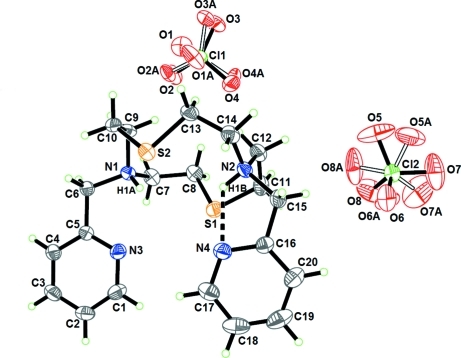
Molecular structure of the title complex with 30% thermal ellipsoids. Dashed line indicate hydrogen bond.

**Fig. 2.**
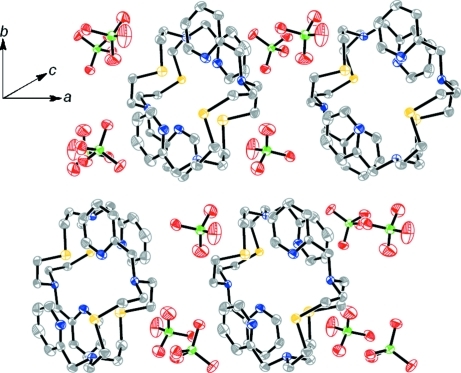
Cell packing of the title complex looking down the c axis, H atoms were ommited for clarity.

## supplementary crystallographic information

### Comment

Macrocyclic ligands have the ability to form transition metal complexes with tunable physicochemical and functional properties (Fabbrizzi *et al.* 1999). The resulting complexes are found to have diverse applications such as with medicinal inorganic compounds, photosensitizers in solar cells, catalysts for organic transformations, molecular devices based on tunable properties, mimics for enzymes catalyzing redox and hydrolytic processes (De Silva *et al.* 2003; Habata *et al.* 2006; Bilgin *et al.* 2009; Bernier *et al.* 2011). Among the large number of known macrocyclic ligands, there has been particular interest in the preparation and characterization of macrocyclic ligands with pendant substituents (Peng *et al.* 2009; Wasitlewski *et al.* 1990). For example, N, S-functionalized macrocycles with pyridyl pendant arms may enhance their metal-ion selectivity depending on their the macrocyclic ring size as well as the pendent moiety. These novel ligands containing the thioether donors as well as aliphatic and pyridyl nitrogen donors, provide a strong mixed nitrogen/sulfur metal coordination environment (Funkemeier *et al.* 1993). In this work, the structure of the title compound with the {py_2_N_2_S_2_}-donor set as a macrocyclic cation is reported.

In the cation, the twelve-membered ring adopts a distorted crown conformation with the sulfur and nitrogen atoms at the point and carbon atoms at the edges (Fig. 1). Due to the preference of C—S bonds to adopt a *gauche* conformation, the sulfur atoms are oriented with their lone pairs pointing out of the ring (Chak *et al.* 2006). The protonated hydrogen atoms bound to the amide atoms are oriented at the inner cavity. The two pyridyl pendent arms are located at the same side, but not parallel to each other with the dihedral angle between the pyridyl rings being 14.61 (13)°. The H1B atom bonded to N2 is involved in a N2—H1B···N4 intramolecular hydrogen bond.  There are no hydrogen bonds between the O atoms of ClO_4_^-^ and the amide H atoms.  The ClO_4_^-^ anions are embedded in the cavity formed by cations (Fig. 2).

### Experimental

A mixture of ligand 4-(pyridin-2-ylmethyl)-1,7-dithia-4,10-diazacyclododecane (1.5 g, 5 mmol) and 2-(chloromethyl)pyridine (0.64 g, 5 mmol) in dry toluene (50 ml) was refluxed under an Ar atmosphere for 48 h in the presence of K_2_CO_3_ (1.40 g, 10.14 mmol) and KI (0.84 g, 5.05 mmol). The resulting orange solution was filtered, and the solvent was removed under reduced pressure. The obtained solid was purified by chromatography using ethyl acetate as the eluant to give a pale yellow solid 4,10-bis(pyridin-2-ylmethyl)-1,7-dithia-4,10-diazacyclododecane, *L* (1.5 g, 79%). Reaction of *L* with Zn(ClO_4_)_2_. 6H_2_O) in MeCN in the presence of little acid afforded a solution. Diffusion of Et_2_O into the MeCN solution gave single crystals of the title product with 25% yield.

### Refinement

In title complex, oxygen atoms in two perchlorate anions exhibited disorder over two positions.  The O(1)—O(4) oxygen atoms bonded to the Cl1 atom, and the O(5)—O(8) oxygen atoms bonded to the Cl2 atom, were split into two fragments with occupancy factors of O(1)—O(4)/O(1 A)—O(4 A) = 0.65 (3)/0.35 (3), and O(5)—O(8)/O(5 A)—O(8 A) = 0.640 (15)/0.360 (15). Hydrogen atoms for the carbon atoms were placed in geometrically idealized positions and constrained to ride on their parent with C—H = 0.97 Å and 0.93 Å for methylene and aryl type H-atoms, respectively, and refined in a riding mode with *U*_iso_(H) = 1.2*U*_eq_(C). The H atoms on the amino atoms were located from the Fourier map and were allowed to refine freely.

### Crystal data

**Table:**

C_20_H_30_N_4_S_2_^2+^·2(ClO_4_^−^)	*Z* = 2
*M**_r_* = 589.52	*F*(000) = 616
Triclinic, *P*1	*D*_x_ = 1.508 Mg m^−^^3^
Hall symbol: -P 1	Mo *K*α radiation, λ = 0.71073 Å
*a* = 10.4491 (6) Å	Cell parameters from 5215 reflections
*b* = 11.6146 (7) Å	θ = 2.4–27.9°
*c* = 11.7795 (7) Å	µ = 0.46 mm^−^^1^
α = 96.167 (1)°	*T* = 296 K
β = 90.340 (1)°	Block, colorless
γ = 113.830 (1)°	0.35 × 0.30 × 0.25 mm
*V* = 1298.23 (13) Å^3^	

### Data collection

**Table:**

Bruker APEXII CCD diffractometer	6516 independent reflections
Radiation source: fine-focus sealed tube	4865 reflections with *I* > 2σ(*I*)
graphite	*R*_int_ = 0.018
Thin slice φ & ω scans	θ_max_ = 28.4°, θ_min_ = 2.2°
Absorption correction: multi-scan (*SADABS*; Sheldrick, 2003)	*h* = −13→12
*T*_min_ = 0.850, *T*_max_ = 0.891	*k* = −15→15
12276 measured reflections	*l* = −15→15

### Refinement

**Table:**

Refinement on *F*^2^	Primary atom site location: structure-invariant direct methods
Least-squares matrix: full	Secondary atom site location: difference Fourier map
*R*[*F*^2^ > 2σ(*F*^2^)] = 0.041	Hydrogen site location: inferred from neighbouring sites
*wR*(*F*^2^) = 0.114	H atoms treated by a mixture of independent and constrained refinement
*S* = 1.04	*w* = 1/[σ^2^(*F*_o_^2^) + (0.0546*P*)^2^ + 0.4486*P*] where *P* = (*F*_o_^2^ + 2*F*_c_^2^)/3
6516 reflections	(Δ/σ)_max_ = 0.001
407 parameters	Δρ_max_ = 0.37 e Å^−^^3^
84 restraints	Δρ_min_ = −0.37 e Å^−^^3^

### Special details

**Table:**

Experimental. Characterization of ligand: 4,10-bis(pyridin-2-ylmethyl)-1,7-dithia-4,10-diazacyclododecane: ^1^H NMR (CDCl_3_): 8.455 (d, J = 4.52, py—H), 7.604 (t, J = 15.08, py—H), 7.475 (d, J = 7.79, py—H), 7.097 (t, J = 12.18, py—H), 3.739 (s, py—CH_2_), 2.802 (t, J = 13.08, NCH_2_CH_2_), 2.729 (t, J = 13.02, NCH_2_CH_2_). ^13^C NMR (CDCl_3_): 159.26, 149.06, 136.50, 123.05, 122.12, 61.35, 52.54, 26.66.
Geometry. All e.s.d.'s (except the e.s.d. in the dihedral angle between two l.s. planes) are estimated using the full covariance matrix. The cell e.s.d.'s are taken into account individually in the estimation of e.s.d.'s in distances, angles and torsion angles; correlations between e.s.d.'s in cell parameters are only used when they are defined by crystal symmetry. An approximate (isotropic) treatment of cell e.s.d.'s is used for estimating e.s.d.'s involving l.s. planes.
Refinement. Refinement of *F*^2^ against ALL reflections. The weighted *R*-factor *wR* and goodness of fit *S* are based on *F*^2^, conventional *R*-factors *R* are based on *F*, with *F* set to zero for negative *F*^2^. The threshold expression of *F*^2^ > σ(*F*^2^) is used only for calculating *R*-factors(gt) *etc*. and is not relevant to the choice of reflections for refinement. *R*-factors based on *F*^2^ are statistically about twice as large as those based on *F*, and *R*- factors based on ALL data will be even larger.

### Fractional atomic coordinates and isotropic or equivalent isotropic displacement parameters (Å^2^)

**Table:**

	*x*	*y*	*z*	*U*_iso_*/*U*_eq_	Occ. (<1)
S1	0.39671 (6)	0.14286 (5)	0.65754 (5)	0.05266 (15)	
S2	0.48251 (5)	0.13448 (5)	1.00606 (5)	0.04540 (14)	
N1	0.21785 (17)	−0.03948 (15)	0.83251 (14)	0.0387 (3)	
N2	0.61643 (17)	0.35034 (16)	0.84963 (16)	0.0444 (4)	
N3	0.34391 (19)	−0.16697 (17)	0.71127 (16)	0.0513 (4)	
N4	0.7232 (2)	0.19581 (18)	0.74762 (17)	0.0541 (4)	
C1	0.4072 (3)	−0.2237 (3)	0.6405 (2)	0.0610 (6)	
H1	0.4842	−0.1727	0.6033	0.073*	
C2	0.3645 (3)	−0.3522 (2)	0.6201 (2)	0.0595 (6)	
H2	0.4120	−0.3872	0.5709	0.071*	
C3	0.2501 (3)	−0.4286 (2)	0.6738 (2)	0.0600 (6)	
H3	0.2181	−0.5164	0.6609	0.072*	
C4	0.1835 (2)	−0.3728 (2)	0.7471 (2)	0.0519 (5)	
H4	0.1064	−0.4221	0.7852	0.062*	
C5	0.23386 (19)	−0.24212 (18)	0.76281 (16)	0.0398 (4)	
C6	0.1675 (2)	−0.17756 (18)	0.84537 (18)	0.0438 (4)	
H6A	0.0665	−0.2179	0.8320	0.053*	
H6B	0.1898	−0.1870	0.9229	0.053*	
C7	0.1455 (2)	−0.0218 (2)	0.7285 (2)	0.0515 (5)	
H7A	0.1414	−0.0853	0.6663	0.062*	
H7B	0.0499	−0.0362	0.7458	0.062*	
C8	0.2161 (2)	0.1076 (2)	0.6893 (2)	0.0532 (5)	
H8A	0.2133	0.1712	0.7485	0.064*	
H8B	0.1655	0.1112	0.6214	0.064*	
C9	0.1998 (2)	0.0353 (2)	0.93856 (18)	0.0469 (5)	
H9A	0.1049	−0.0067	0.9622	0.056*	
H9B	0.2121	0.1186	0.9207	0.056*	
C10	0.3007 (2)	0.05079 (19)	1.03664 (17)	0.0460 (5)	
H10A	0.2865	−0.0324	1.0561	0.055*	
H10B	0.2800	0.0965	1.1027	0.055*	
C11	0.4678 (3)	0.3147 (2)	0.6720 (2)	0.0606 (6)	
H11A	0.5516	0.3453	0.6291	0.073*	
H11B	0.4001	0.3403	0.6381	0.073*	
C12	0.5040 (2)	0.3773 (2)	0.7943 (2)	0.0543 (5)	
H12A	0.5350	0.4682	0.7953	0.065*	
H12B	0.4207	0.3470	0.8378	0.065*	
C13	0.5041 (2)	0.29796 (19)	1.03629 (19)	0.0521 (5)	
H13A	0.4205	0.3061	1.0094	0.063*	
H13B	0.5174	0.3235	1.1182	0.063*	
C14	0.6293 (2)	0.3825 (2)	0.9775 (2)	0.0533 (5)	
H14A	0.6412	0.4698	0.9958	0.064*	
H14B	0.7125	0.3757	1.0069	0.064*	
C15	0.7559 (2)	0.4119 (2)	0.7978 (2)	0.0571 (6)	
H15A	0.8295	0.4504	0.8579	0.069*	
H15B	0.7552	0.4780	0.7543	0.069*	
C16	0.7840 (2)	0.3135 (2)	0.72044 (18)	0.0479 (5)	
C17	0.7505 (3)	0.1055 (3)	0.6860 (2)	0.0642 (6)	
H17	0.7076	0.0225	0.7034	0.077*	
C18	0.8396 (3)	0.1311 (3)	0.5980 (2)	0.0791 (9)	
H18	0.8592	0.0671	0.5581	0.095*	
C19	0.8991 (3)	0.2522 (4)	0.5698 (2)	0.0868 (10)	
H19	0.9586	0.2712	0.5096	0.104*	
C20	0.8703 (3)	0.3455 (3)	0.6311 (2)	0.0677 (7)	
H20	0.9082	0.4282	0.6126	0.081*	
Cl1	0.07973 (5)	0.30206 (4)	0.92855 (4)	0.04391 (13)	
O1	0.1496 (11)	0.2960 (8)	1.0304 (5)	0.0704 (15)	0.65 (3)
O2	−0.0140 (9)	0.1779 (8)	0.8848 (8)	0.0671 (19)	0.65 (3)
O3	0.0093 (11)	0.3821 (9)	0.9534 (8)	0.0776 (19)	0.65 (3)
O4	0.1840 (8)	0.3539 (7)	0.8490 (7)	0.077 (2)	0.65 (3)
O1A	0.1935 (18)	0.3149 (12)	1.003 (2)	0.082 (5)	0.35 (3)
O2A	−0.0087 (13)	0.1694 (11)	0.9035 (16)	0.057 (3)	0.35 (3)
O3A	0.0013 (17)	0.3660 (14)	0.9828 (18)	0.075 (4)	0.35 (3)
O4A	0.130 (3)	0.3533 (14)	0.8256 (12)	0.106 (5)	0.35 (3)
Cl2	0.77446 (5)	0.71112 (5)	0.63800 (4)	0.04936 (14)	
O5	0.7051 (8)	0.7250 (11)	0.7361 (3)	0.143 (5)	0.640 (15)
O6	0.8883 (4)	0.6809 (5)	0.6678 (5)	0.074 (2)	0.640 (15)
O7	0.8261 (6)	0.8223 (5)	0.5868 (9)	0.136 (4)	0.640 (15)
O8	0.6782 (6)	0.6138 (6)	0.5611 (5)	0.111 (3)	0.640 (15)
O5A	0.7899 (12)	0.8133 (10)	0.7147 (11)	0.124 (5)	0.360 (15)
O6A	0.8869 (9)	0.6775 (10)	0.6571 (9)	0.086 (5)	0.360 (15)
O7A	0.7803 (15)	0.7432 (18)	0.5294 (7)	0.152 (8)	0.360 (15)
O8A	0.6522 (6)	0.6099 (9)	0.6511 (16)	0.146 (10)	0.360 (15)
H1A	0.304 (2)	−0.011 (2)	0.8194 (19)	0.047 (6)*	
H1B	0.594 (2)	0.268 (2)	0.8319 (19)	0.054 (6)*	

### Atomic displacement parameters (Å^2^)

**Table:**

	*U*^11^	*U*^22^	*U*^33^	*U*^12^	*U*^13^	*U*^23^
S1	0.0584 (3)	0.0548 (3)	0.0439 (3)	0.0224 (3)	0.0024 (2)	0.0043 (2)
S2	0.0438 (3)	0.0413 (3)	0.0502 (3)	0.0173 (2)	−0.0050 (2)	0.0018 (2)
N1	0.0315 (8)	0.0370 (8)	0.0453 (9)	0.0132 (6)	−0.0011 (7)	−0.0012 (7)
N2	0.0392 (9)	0.0323 (8)	0.0586 (11)	0.0122 (7)	0.0048 (7)	0.0023 (7)
N3	0.0492 (10)	0.0452 (9)	0.0525 (10)	0.0128 (8)	0.0094 (8)	0.0020 (8)
N4	0.0549 (11)	0.0486 (10)	0.0573 (11)	0.0211 (9)	0.0074 (9)	−0.0002 (8)
C1	0.0543 (13)	0.0687 (15)	0.0564 (14)	0.0219 (12)	0.0157 (11)	0.0039 (12)
C2	0.0682 (15)	0.0702 (15)	0.0518 (13)	0.0427 (13)	0.0046 (11)	−0.0035 (11)
C3	0.0730 (16)	0.0469 (12)	0.0644 (15)	0.0312 (12)	0.0016 (12)	−0.0018 (11)
C4	0.0514 (12)	0.0419 (11)	0.0581 (13)	0.0150 (9)	0.0062 (10)	0.0043 (9)
C5	0.0371 (9)	0.0387 (9)	0.0408 (10)	0.0136 (8)	−0.0026 (8)	0.0004 (8)
C6	0.0413 (10)	0.0366 (9)	0.0485 (11)	0.0113 (8)	0.0059 (8)	0.0023 (8)
C7	0.0433 (11)	0.0504 (12)	0.0571 (13)	0.0170 (9)	−0.0137 (9)	0.0002 (10)
C8	0.0517 (12)	0.0557 (13)	0.0560 (13)	0.0262 (10)	−0.0088 (10)	0.0051 (10)
C9	0.0407 (10)	0.0459 (11)	0.0560 (12)	0.0220 (9)	0.0065 (9)	−0.0047 (9)
C10	0.0532 (12)	0.0428 (10)	0.0397 (10)	0.0182 (9)	0.0102 (9)	0.0003 (8)
C11	0.0657 (15)	0.0578 (13)	0.0571 (14)	0.0208 (11)	−0.0007 (11)	0.0194 (11)
C12	0.0530 (12)	0.0455 (11)	0.0679 (15)	0.0236 (10)	−0.0001 (11)	0.0076 (10)
C13	0.0576 (13)	0.0412 (11)	0.0509 (12)	0.0167 (9)	0.0003 (10)	−0.0085 (9)
C14	0.0490 (12)	0.0411 (11)	0.0590 (13)	0.0107 (9)	−0.0052 (10)	−0.0081 (9)
C15	0.0444 (11)	0.0405 (11)	0.0772 (16)	0.0078 (9)	0.0129 (11)	0.0068 (10)
C16	0.0371 (10)	0.0536 (12)	0.0489 (12)	0.0151 (9)	0.0006 (9)	0.0020 (9)
C17	0.0759 (17)	0.0615 (15)	0.0601 (15)	0.0366 (13)	−0.0060 (13)	−0.0072 (12)
C18	0.0822 (19)	0.105 (2)	0.0592 (16)	0.0554 (19)	−0.0064 (14)	−0.0240 (16)
C19	0.0719 (19)	0.119 (3)	0.0550 (16)	0.0290 (19)	0.0155 (14)	−0.0095 (17)
C20	0.0587 (15)	0.0762 (17)	0.0548 (14)	0.0143 (13)	0.0084 (11)	0.0054 (12)
Cl1	0.0434 (3)	0.0362 (2)	0.0537 (3)	0.01821 (19)	0.0054 (2)	0.00373 (19)
O1	0.068 (4)	0.084 (3)	0.060 (3)	0.034 (3)	−0.012 (2)	0.0022 (18)
O2	0.057 (3)	0.047 (3)	0.086 (3)	0.012 (2)	−0.016 (2)	−0.003 (2)
O3	0.088 (4)	0.071 (3)	0.101 (4)	0.060 (3)	0.009 (3)	0.005 (3)
O4	0.076 (3)	0.060 (2)	0.079 (3)	0.0132 (19)	0.033 (2)	0.003 (2)
O1A	0.050 (6)	0.044 (4)	0.148 (11)	0.021 (4)	−0.039 (6)	−0.013 (5)
O2A	0.040 (5)	0.033 (4)	0.098 (8)	0.013 (3)	0.009 (5)	0.007 (4)
O3A	0.097 (6)	0.057 (4)	0.091 (8)	0.049 (4)	0.029 (5)	0.014 (5)
O4A	0.151 (14)	0.085 (6)	0.086 (6)	0.043 (8)	0.051 (7)	0.051 (5)
Cl2	0.0486 (3)	0.0477 (3)	0.0509 (3)	0.0183 (2)	−0.0043 (2)	0.0072 (2)
O5	0.141 (7)	0.260 (13)	0.065 (2)	0.127 (9)	0.025 (3)	−0.008 (4)
O6	0.054 (3)	0.069 (4)	0.092 (4)	0.015 (3)	−0.022 (3)	0.026 (3)
O7	0.135 (4)	0.090 (4)	0.179 (9)	0.028 (3)	−0.035 (5)	0.073 (4)
O8	0.091 (4)	0.101 (5)	0.120 (5)	0.035 (3)	−0.048 (3)	−0.053 (4)
O5A	0.108 (7)	0.107 (7)	0.154 (10)	0.055 (6)	0.017 (7)	−0.047 (7)
O6A	0.070 (8)	0.124 (11)	0.099 (8)	0.070 (7)	0.039 (6)	0.035 (7)
O7A	0.176 (14)	0.24 (2)	0.073 (6)	0.104 (16)	−0.007 (6)	0.081 (8)
O8A	0.039 (3)	0.096 (8)	0.30 (3)	0.005 (4)	0.001 (7)	0.101 (13)

### Geometric parameters (Å, °)

**Table:**

S1—C8	1.813 (2)	C11—C12	1.514 (3)
S1—C11	1.814 (2)	C11—H11A	0.9700
S2—C10	1.810 (2)	C11—H11B	0.9700
S2—C13	1.814 (2)	C12—H12A	0.9700
N1—C6	1.498 (2)	C12—H12B	0.9700
N1—C9	1.501 (3)	C13—C14	1.508 (3)
N1—C7	1.511 (3)	C13—H13A	0.9700
N1—H1A	0.85 (2)	C13—H13B	0.9700
N2—C12	1.495 (3)	C14—H14A	0.9700
N2—C14	1.504 (3)	C14—H14B	0.9700
N2—C15	1.507 (3)	C15—C16	1.508 (3)
N2—H1B	0.89 (2)	C15—H15A	0.9700
N3—C5	1.328 (3)	C15—H15B	0.9700
N3—C1	1.344 (3)	C16—C20	1.373 (3)
N4—C16	1.330 (3)	C17—C18	1.373 (4)
N4—C17	1.338 (3)	C17—H17	0.9300
C1—C2	1.367 (3)	C18—C19	1.368 (5)
C1—H1	0.9300	C18—H18	0.9300
C2—C3	1.374 (4)	C19—C20	1.373 (4)
C2—H2	0.9300	C19—H19	0.9300
C3—C4	1.378 (3)	C20—H20	0.9300
C3—H3	0.9300	Cl1—O3	1.410 (6)
C4—C5	1.381 (3)	Cl1—O2	1.413 (7)
C4—H4	0.9300	Cl1—O4A	1.418 (10)
C5—C6	1.503 (3)	Cl1—O1A	1.422 (10)
C6—H6A	0.9700	Cl1—O1	1.423 (6)
C6—H6B	0.9700	Cl1—O3A	1.424 (11)
C7—C8	1.508 (3)	Cl1—O4	1.425 (6)
C7—H7A	0.9700	Cl1—O2A	1.437 (11)
C7—H7B	0.9700	Cl2—O5A	1.365 (6)
C8—H8A	0.9700	Cl2—O7A	1.365 (6)
C8—H8B	0.9700	Cl2—O8A	1.366 (5)
C9—C10	1.505 (3)	Cl2—O7	1.389 (4)
C9—H9A	0.9700	Cl2—O5	1.396 (4)
C9—H9B	0.9700	Cl2—O8	1.400 (4)
C10—H10A	0.9700	Cl2—O6A	1.403 (7)
C10—H10B	0.9700	Cl2—O6	1.421 (4)
			
C8—S1—C11	100.22 (12)	H13A—C13—H13B	108.2
C10—S2—C13	101.11 (10)	N2—C14—C13	113.20 (17)
C6—N1—C9	111.86 (16)	N2—C14—H14A	108.9
C6—N1—C7	110.19 (15)	C13—C14—H14A	108.9
C9—N1—C7	111.66 (16)	N2—C14—H14B	108.9
C6—N1—H1A	108.5 (15)	C13—C14—H14B	108.9
C9—N1—H1A	108.7 (15)	H14A—C14—H14B	107.8
C7—N1—H1A	105.7 (15)	N2—C15—C16	109.51 (17)
C12—N2—C14	112.94 (17)	N2—C15—H15A	109.8
C12—N2—C15	112.52 (18)	C16—C15—H15A	109.8
C14—N2—C15	111.17 (17)	N2—C15—H15B	109.8
C12—N2—H1B	106.4 (15)	C16—C15—H15B	109.8
C14—N2—H1B	109.7 (15)	H15A—C15—H15B	108.2
C15—N2—H1B	103.5 (15)	N4—C16—C20	123.1 (2)
C5—N3—C1	116.90 (19)	N4—C16—C15	115.22 (19)
C16—N4—C17	118.0 (2)	C20—C16—C15	121.6 (2)
N3—C1—C2	123.6 (2)	N4—C17—C18	122.2 (3)
N3—C1—H1	118.2	N4—C17—H17	118.9
C2—C1—H1	118.2	C18—C17—H17	118.9
C1—C2—C3	118.7 (2)	C19—C18—C17	119.1 (3)
C1—C2—H2	120.6	C19—C18—H18	120.5
C3—C2—H2	120.6	C17—C18—H18	120.5
C2—C3—C4	118.8 (2)	C18—C19—C20	119.4 (3)
C2—C3—H3	120.6	C18—C19—H19	120.3
C4—C3—H3	120.6	C20—C19—H19	120.3
C3—C4—C5	118.6 (2)	C16—C20—C19	118.2 (3)
C3—C4—H4	120.7	C16—C20—H20	120.9
C5—C4—H4	120.7	C19—C20—H20	120.9
N3—C5—C4	123.37 (19)	O3—Cl1—O2	111.7 (3)
N3—C5—C6	116.43 (17)	O3—Cl1—O4A	93.2 (8)
C4—C5—C6	120.16 (18)	O2—Cl1—O4A	100.5 (9)
N1—C6—C5	111.39 (16)	O3—Cl1—O1A	119.0 (9)
N1—C6—H6A	109.4	O2—Cl1—O1A	117.5 (7)
C5—C6—H6A	109.4	O4A—Cl1—O1A	110.3 (5)
N1—C6—H6B	109.4	O3—Cl1—O1	109.2 (3)
C5—C6—H6B	109.4	O2—Cl1—O1	108.7 (3)
H6A—C6—H6B	108.0	O4A—Cl1—O1	132.2 (10)
C8—C7—N1	113.97 (16)	O2—Cl1—O3A	108.6 (10)
C8—C7—H7A	108.8	O4A—Cl1—O3A	109.5 (5)
N1—C7—H7A	108.8	O1A—Cl1—O3A	110.0 (5)
C8—C7—H7B	108.8	O1—Cl1—O3A	96.1 (9)
N1—C7—H7B	108.8	O3—Cl1—O4	109.7 (3)
H7A—C7—H7B	107.7	O2—Cl1—O4	110.1 (3)
C7—C8—S1	111.49 (15)	O1A—Cl1—O4	85.3 (9)
C7—C8—H8A	109.3	O1—Cl1—O4	107.4 (3)
S1—C8—H8A	109.3	O3A—Cl1—O4	124.4 (7)
C7—C8—H8B	109.3	O3—Cl1—O2A	115.5 (9)
S1—C8—H8B	109.3	O4A—Cl1—O2A	109.6 (5)
H8A—C8—H8B	108.0	O1A—Cl1—O2A	108.1 (5)
N1—C9—C10	113.65 (16)	O1—Cl1—O2A	98.1 (8)
N1—C9—H9A	108.8	O3A—Cl1—O2A	109.4 (5)
C10—C9—H9A	108.8	O4—Cl1—O2A	115.9 (8)
N1—C9—H9B	108.8	O5A—Cl2—O7A	109.7 (5)
C10—C9—H9B	108.8	O5A—Cl2—O8A	111.1 (5)
H9A—C9—H9B	107.7	O7A—Cl2—O8A	111.1 (5)
C9—C10—S2	113.47 (14)	O5A—Cl2—O7	69.0 (5)
C9—C10—H10A	108.9	O8A—Cl2—O7	141.3 (4)
S2—C10—H10A	108.9	O5A—Cl2—O5	47.2 (4)
C9—C10—H10B	108.9	O7A—Cl2—O5	136.7 (5)
S2—C10—H10B	108.9	O8A—Cl2—O5	65.0 (5)
H10A—C10—H10B	107.7	O7—Cl2—O5	110.9 (3)
C12—C11—S1	114.19 (15)	O5A—Cl2—O8	143.4 (4)
C12—C11—H11A	108.7	O7A—Cl2—O8	67.6 (6)
S1—C11—H11A	108.7	O8A—Cl2—O8	46.7 (6)
C12—C11—H11B	108.7	O7—Cl2—O8	109.0 (3)
S1—C11—H11B	108.7	O5—Cl2—O8	108.0 (3)
H11A—C11—H11B	107.6	O5A—Cl2—O6A	108.4 (5)
N2—C12—C11	111.90 (19)	O7A—Cl2—O6A	107.7 (4)
N2—C12—H12A	109.2	O8A—Cl2—O6A	108.7 (4)
C11—C12—H12A	109.2	O7—Cl2—O6A	107.5 (5)
N2—C12—H12B	109.2	O5—Cl2—O6A	114.3 (5)
C11—C12—H12B	109.2	O8—Cl2—O6A	106.9 (5)
H12A—C12—H12B	107.9	O5A—Cl2—O6	104.1 (5)
C14—C13—S2	109.68 (15)	O7A—Cl2—O6	112.2 (6)
C14—C13—H13A	109.7	O8A—Cl2—O6	108.6 (4)
S2—C13—H13A	109.7	O7—Cl2—O6	108.8 (3)
C14—C13—H13B	109.7	O5—Cl2—O6	109.5 (3)
S2—C13—H13B	109.7	O8—Cl2—O6	110.6 (3)
			
C5—N3—C1—C2	−0.3 (4)	C8—S1—C11—C12	80.8 (2)
N3—C1—C2—C3	0.5 (4)	C14—N2—C12—C11	−165.53 (18)
C1—C2—C3—C4	−0.7 (4)	C15—N2—C12—C11	67.6 (2)
C2—C3—C4—C5	0.6 (4)	S1—C11—C12—N2	62.8 (2)
C1—N3—C5—C4	0.2 (3)	C10—S2—C13—C14	−159.75 (16)
C1—N3—C5—C6	177.6 (2)	C12—N2—C14—C13	67.5 (2)
C3—C4—C5—N3	−0.4 (3)	C15—N2—C14—C13	−164.93 (18)
C3—C4—C5—C6	−177.7 (2)	S2—C13—C14—N2	60.1 (2)
C9—N1—C6—C5	−157.83 (16)	C12—N2—C15—C16	−103.4 (2)
C7—N1—C6—C5	77.3 (2)	C14—N2—C15—C16	128.8 (2)
N3—C5—C6—N1	13.8 (2)	C17—N4—C16—C20	1.5 (3)
C4—C5—C6—N1	−168.68 (18)	C17—N4—C16—C15	−176.4 (2)
C6—N1—C7—C8	−163.84 (18)	N2—C15—C16—N4	−27.6 (3)
C9—N1—C7—C8	71.2 (2)	N2—C15—C16—C20	154.5 (2)
N1—C7—C8—S1	57.5 (2)	C16—N4—C17—C18	0.9 (4)
C11—S1—C8—C7	−157.69 (17)	N4—C17—C18—C19	−2.1 (4)
C6—N1—C9—C10	72.1 (2)	C17—C18—C19—C20	1.0 (5)
C7—N1—C9—C10	−163.87 (17)	N4—C16—C20—C19	−2.5 (4)
N1—C9—C10—S2	61.1 (2)	C15—C16—C20—C19	175.2 (2)
C13—S2—C10—C9	83.22 (16)	C18—C19—C20—C16	1.2 (4)

### Hydrogen-bond geometry (Å, °)

**Table:**

*D*—H···*A*	*D*—H	H···*A*	*D*···*A*	*D*—H···*A*
N2—H1B···N4	0.89 (2)	2.06 (2)	2.661 (3)	123.6 (19)
